# Integrating historical clinical and financial data for pharmacological research

**DOI:** 10.1186/1471-2288-11-151

**Published:** 2011-11-18

**Authors:** Vikrant G Deshmukh, N Brett Sower, Cheri Y Hunter, Joyce A Mitchell

**Affiliations:** 1 Department of Biomedical Informatics, School of Medicine, University of Utah, 26 South 2000 East Room 5775, Salt Lake City, UT 84112 USA; 2 Information Technology Services, University of Utah Healthcare, 585 Komas Drive, Salt Lake City, UT 84108 USA; 3 Pharmacy Services, University of Utah Healthcare, 50 South Medical Drive, Salt Lake City, UT 84132 USA

## Abstract

**Background:**

Retrospective research requires longitudinal data, and repositories derived from electronic health records (EHR) can be sources of such data. With Health Information Technology for Economic and Clinical Health (HITECH) Act meaningful use provisions, many institutions are expected to adopt EHRs, but may be left with large amounts of financial and historical clinical data, which can differ significantly from data obtained from newer systems, due to lack or inconsistent use of controlled medical terminologies (CMT) in older systems. We examined different approaches for semantic enrichment of financial data with CMT, and integration of clinical data from disparate historical and current sources for research.

**Methods:**

Snapshots of financial data from 1999, 2004 and 2009 were mapped automatically to the current inpatient pharmacy catalog, and enriched with RxNorm. Administrative metadata from financial and dispensing systems, RxNorm and two commercial pharmacy vocabularies were used to integrate data from current and historical inpatient pharmacy modules, and the outpatient EHR. Data integration approaches were compared using percentages of automated matches, and effects on cohort size of a retrospective study.

**Results:**

During 1999-2009, 71.52%-90.08% of items in use from the financial catalog were enriched using RxNorm; 64.95%-70.37% of items in use from the historical inpatient system were integrated using RxNorm, 85.96%-91.67% using a commercial vocabulary, 87.19%-94.23% using financial metadata, and 77.20%-94.68% using dispensing metadata. During 1999-2009, 48.01%-30.72% of items in use from the outpatient catalog were integrated using RxNorm, and 79.27%-48.60% using a commercial vocabulary. In a cohort of 16304 inpatients obtained from clinical systems, 4172 (25.58%) were found exclusively through integration of historical clinical data, while 15978 (98%) could be identified using semantically enriched financial data.

**Conclusions:**

Data integration using metadata from financial/dispensing systems and pharmacy vocabularies were comparable. Given the current state of EHR adoption, semantic enrichment of financial data and integration of historical clinical data would allow the repurposing of these data for research. With the push for HITECH meaningful use, institutions that are transitioning to newer EHRs will be able to use their older financial and clinical data for research using these methods.

## Background

Early Electronic Health Record (EHR) systems were electronic versions of traditional, paper-based medical records, used in medicine. The financial and administrative portions of the medical record were computerized first [1], and specialized hospital information systems gradually evolved into the first, comprehensive, modern EHRs [2,3]. Rapid developments in information technology and growth in computing power have led to the development of EHR systems that surpass their predecessors in functionality and complexity. These changes coincided with improvements in controlled medical terminologies (CMT) [4], and standards for data storage, representation & exchange. CMT in modern EHR systems allow for precise semantic definitions that were not possible in many historical systems. The adoption of EHR systems in an inpatient setting has been slower than expected [5,6], whereas specialized hospital financial systems remain pervasive [7]. With the *meaningful use *provisions of the Health Information Technology for Economic and Clinical Health (HITECH) act of 2009, more institutions are expected to adopt modern EHR systems [8]. In spite of these developments, many institutions will continue to have several years' worth of financial or historical clinical data, which can differ significantly from data obtained from modern EHR systems.

There is a growing interest in the secondary use and sharing of EHR data for research; several frameworks are available for this purpose, and CMT are key enablers [9-11]. A majority of EHR systems use commercial pharmacy vocabularies, which have many [12], but not all desirable characteristics of a CMT [4], although most of them map to RxNorm [13]. RxNorm is a CMT of drugs and devices, one of the recommended national standards for pharmacy data, and contains a semantic network of concepts and relationships. Semantic enrichment approaches--in which semantic contextual information is added to data or metadata--have been applied successfully in information retrieval for enhancement of textual documents, in clinical decision support systems like InfoButtons, in natural language processing for annotating unstructured notes, in the development of medical ontologies, etc [14-17]. Mapping local terminologies to CMT like RxNorm can allow semantic enrichment by the extension of semantic attributes to these terminologies [13]. The integration of pharmacy data from different EHR systems [18-20], as well as enrichment of financial data with CMT, can allow for semantic normalization and consistent use of such data in research, and provide long-term value to institutions that have large repositories of such data.

Financial and billing data can be represented by vocabularies like the International Classification of Diseases Ninth Revision, Clinical Modifications [21] (ICD9-CM) for diagnoses, Current Procedural Terminology [22] (CPT) for procedures, diagnosis related groups (DRG), etc.; however, each of these coding systems by themselves lack the level of detail required for clinical care and research [23]. In the pharmacy domain, Healthcare Common Procedure Coding System [24] (HCPCS) is also used for coding certain medication data in the financial system, although HCPCS codes may not be available for all medications, and are not granular enough to distinguish between different doses, routes and forms of a given drug, making them also unsuitable for clinical care and research. In spite of these limitations, data from financial systems have been used for epidemiological research, particularly for selection of patient cohorts based on demographics, diagnoses and procedures. In a previous study, medications were the fourth most common type of inclusion criteria among data requested by researchers [25]. Semantic enrichment of financial data would allow their use in cohort selection, in addition to demographics, diagnoses and procedures, which can be already obtained from financial systems. However, unlike diagnoses and procedures, where ICD9-CM and CPT have been respectively used in coding for several years, RxNorm--the US national standard for medications--has not yet been as widely adopted in EHR systems. In order to study the feasibility of using historical clinical and semantically enriched financial medication data for research, it is necessary to develop strategies for data integration.

In the present investigation, we develop strategies for semantic enrichment of financial data with RxNorm, and compare two different automated approaches for the integration of medication data from disparate clinical systems. Under the first approach for data integration, we use administrative metadata [26,27] from the financial system and the medication dispensing system, to create automated crosswalks between systems. Under the second approach, we use RxNorm and two different commercial pharmacy vocabularies for data integration. We also compare the sensitivity of data integration approaches by using the percentage of matches to the current inpatient system as a metric. The purpose of developing automated matching methods is to create an initial population of vocabulary matches across different systems, which can be reviewed by a terminology expert. We begin with a description of various systems, and how clinical and financial data related to medications are captured electronically at our institution. We then describe the relevant metadata and vocabularies available in each system, and methods for mapping between various systems using metadata and vocabularies. Finally, we evaluate the effect of enriched financial data on the cohort size in an IRB-approved clinical study, and discuss the challenges faced during enrichment and integration of historical data in the context of modern EHRs.

## Methods

### Data sources and preparation

**Figure 1:**
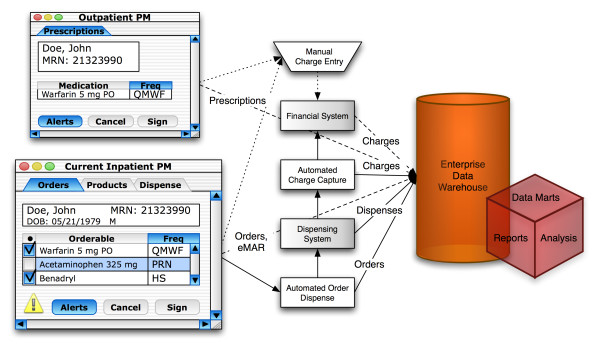
**Data flow in pharmacy**. A simplified view of pharmacy data flow between the inpatient and outpatient EHRs, the financial and dispensing systems, and the Enterprise Data Warehouse (EDW). Batch processes are indicated by dashed lines, Health Level Seven (HL7) processes are indicated with solid lines, while manual processes are indicated with dotted lines

A simplified overview of inpatient and outpatient clinical systems that contain pharmacy data, and their relationship with the financial information system as well as the Enterprise Data Warehouse [26] (EDW) at the University of Utah is shown in Figure 1. Patient-level pharmacy financial data were maintained in the Allegra financial system (IntraNexus Corp., Virginia Beach, VA) until late 2010, when this system was supplanted by Epic for Business (Epic Systems, Verona, WI). Inpatient formulary management and patient-level medication order data were managed within Cerner PharmNet (Cerner Corporation, Kansas City, MO) pharmacy module (PM) and Computerized Provider Order Entry (CPOE; 2009 onwards) modules, which account for over 80% of all medication orders. Drug dispenses are managed in the OmniCell (OmniCell Inc. Mountain View, CA) dispensing system. The inpatient EHR contains electronic Medication Administration Records (eMAR) data since June 2007, which are also available in the EDW. Medication orders data can also originate in other ancillary systems in the inpatient setting, or in the EpicCare Ambulatory EHR system (Epic Systems Corporation, Verona, WI). A majority of clinical systems have automated charge capture process, which allows posting financial transactions directly to the financial systems through various Health Level Seven [28] (HL7) interfaces. A few systems rely on post-hoc manual charge entry, which is also available in the automated systems. The historical inpatient PM had similar data flows to the current inpatient EHR.

Our institution has had several different inpatient EHR systems and two different PMs since 1993. As shown in Table 1 pharmacy data from current inpatient and outpatient EHR systems have been available since 2003 and 1995 respectively; data from historical inpatient PM as well as the financial system have been available since 1999 in the EDW. In addition, several ancillary systems contain pharmacy data, including the radiology system (contrast dyes, etc.), the anesthesiology and surgery systems (anesthetics, antibiotics, etc.), which are also available in the EDW. All of the data were obtained from the EDW. Given the history of various clinical and financial systems between 1993 and 2010, a total of 3 time-points (1999, 2004 and 2009) were selected for further analysis of the dictionary/reference data, since patient-level pharmacy data prior to 1999 were not available in the EDW. A new inpatient EHR was introduced in 1999, followed by another one in mid-2003. CPOE was introduced in the inpatient EHR in 2009, and it has since supplanted the inpatient PM as the principal method of entering medication orders. In order to perform the integration and enrichment processes described below, the medication catalogs/formularies (clinical reference), financial catalog (financial reference), patient-level clinical records (clinical activity) and patient-level financial records (financial activity) data were extracted from the above systems in the EDW for each of the calendar years mentioned above, where such data were available.

**Table 1:** Sources of pharmacy data

		Financial		Historical Clinical		Current Clinical	
		
Type		1993 to 2010	2010 to date	1993 to 1999	1999 to 2003	2003 to date	1995 to date
EHR/Financial System		Allegra	Epic for Business	OACIS	e-Chart	Cerner Millennium Inpatient	EpicCare Ambulatory

Pharmacy Module		-	-	MS Meds		PharmNet, CPOE	CPOE

Automated pharmacy charges		✓	✓		✓	✓	

Manual pharmacy charges		✓	✓	✓	✓	✓	✓

Pharmacy data in the EDW		1999 onwards	✓		✓	✓	✓

Metadata	Financial (charge codes)	✓	✓	✓	✓	✓	
	
	Dispensing (dispense codes)				✓	✓	

Vocabularies	NDC [32]		✓	✓	✓	✓	✓
	
	Multum [30]			✓	✓	✓	
	
	MediSpan [31]						✓

A list of financial & clinical systems that contain pharmacy data, their availability in the EDW, and the type of metadata and vocabularies available for each system.

### Data integration and enrichment

In the heterogeneous EHR infrastructure consisting primarily of commercially developed systems, metadata and vocabulary were managed under a decentralized model, and individual systems were identified as the sources of truth for specific attributes as part of data scrubbing [29]. The EDW served as the source for extracting reference data from individual systems as well as commercial vocabularies and CMT (RxNorm). The current inpatient EHR as well as the historical PM had supported Cerner Multum [30] (Cerner Corporation, Kansas City, MO), although the last snapshot from the historical PM before it was decommissioned did not include these codes, and they were unavailable during the integration process. The current outpatient EHR supported Wolters Kluwer MediSpan [31] (Wolters Kluwer Health, Indianapolis, IN), which was not used in any of the other systems. Orderables in each of the three clinical systems also contained National Drug Code [32] (NDC) as one of the attributes.

**Figure 2:**
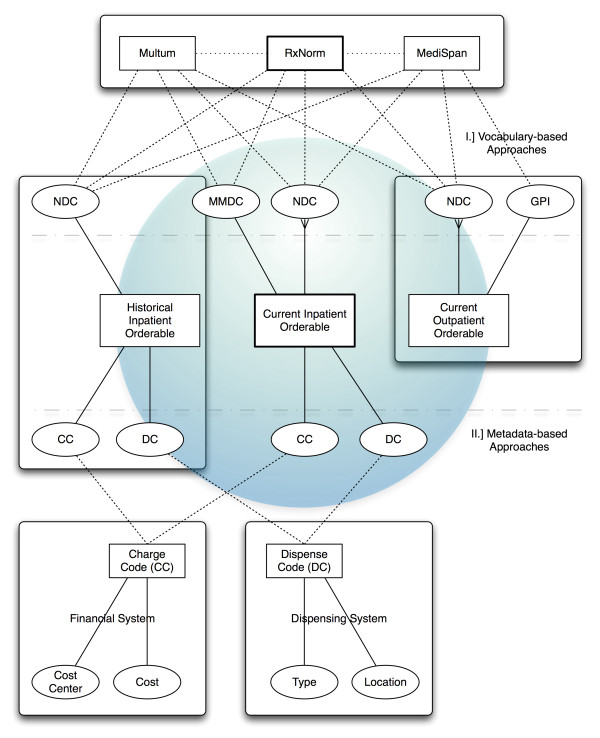
**Approaches to data integration**. Vocabulary- and metadata-based approaches to data integration with the current inpatient system serving as a hub. Integration and enrichment of financial data relied on initial matching to the inpatient orderable using charge codes. Subsequent enrichment, as well as integration between the systems was performed using various methods

Two types of methods were used in data-integration, depending on the types of attributes available. The current inpatient system, which contained the maximum number of current vocabulary and administrative metadata [26,27] attributes served as the hub for integrating data between different systems (Figure 2). The financial system (FS) served as the source of truth for *charge *codes (CC) which were entities in the FS; the dispensing system (DS) serves as the source of truth for *dispense *codes (DC) which are entities within the DS. Both charge and dispense codes were referenced as metadata attributes within the current inpatient EHR, the historical PM, as well as any other system that supports automated charge capture (Figure 1). Other detailed attributes for charge and dispense codes were obtained from the respective source systems. Exact matching of entities and/or attributes that were common among the different systems was used to create crosswalks between the different systems. The crosswalks were then used to generate an 'ontological match' between the different systems, although it is important to note that the financial system as well as the historical inpatient pharmacy module did not have true ontologies, unlike the modern system, which is mapped to a CMT.

### Enrichment of financial data

**Figure 3:**
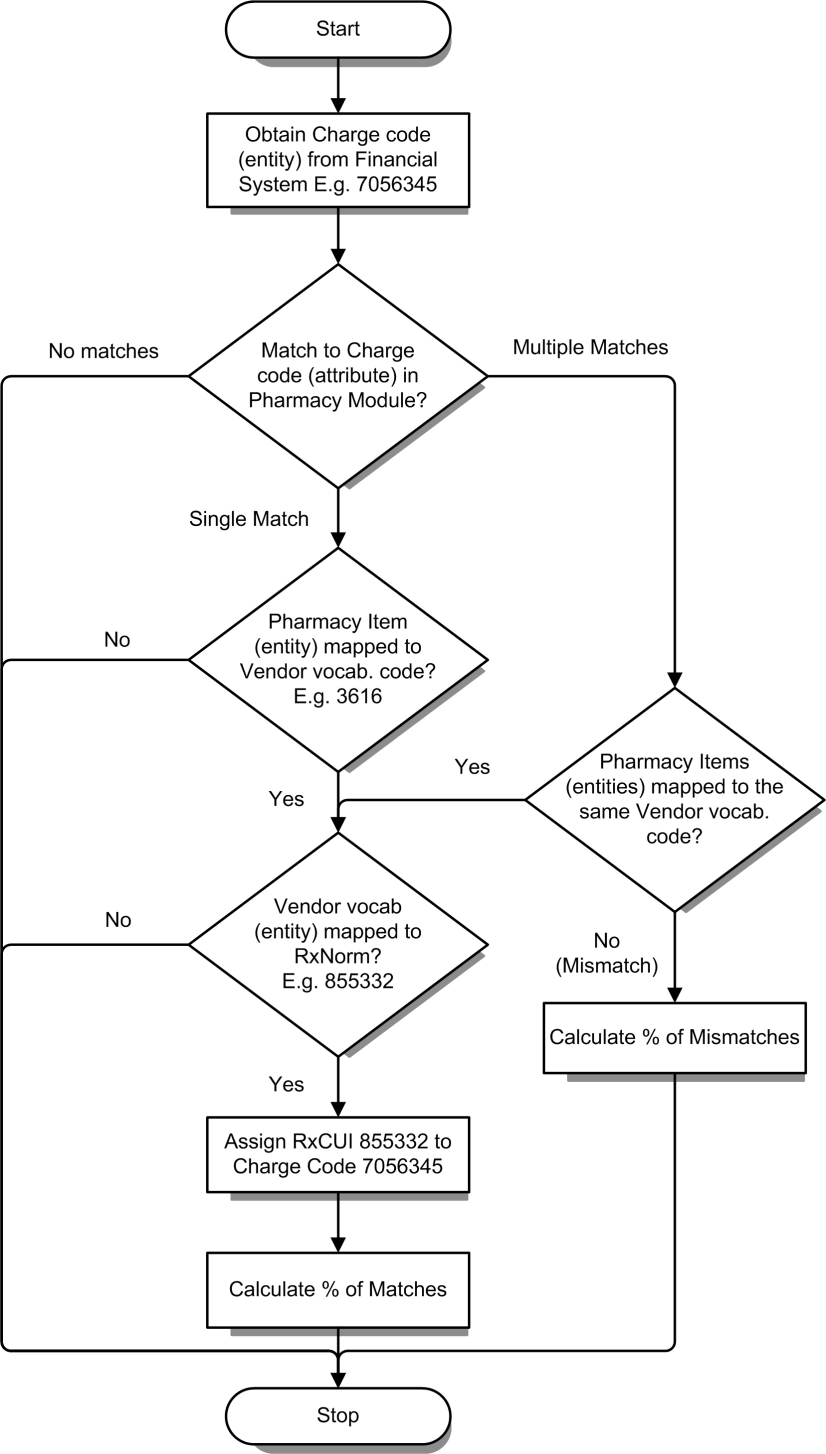
**Mapping and semantic enrichment of financial data**. An example of code-matching between the financial system and the current inpatient pharmacy module, with links to RxNorm. *Warfarin, 5 mg oral tablet *in the financial system had a charge code of *7056345*, which matched to a single item in the current inpatient pharmacy module. This item had the main Multum drug code (MMDC) *3616*, which mapped to RxNorm code *855332*, which could now be assigned to the charge code. Matching and enrichment of financial codes using RxNorm allowed for queries in the financial data using RxNorm.

Enrichment of financial data with CMT was performed in two steps. An initial match was performed between items in the financial system and orderables in the current inpatient EHR using the metadata *attribute *charge code of the inpatient orderable, where such links existed. Links from the clinical system to RxNorm (release May 3, 2010 was available in the EDW at the time of analysis) were then used to semantically enrich the charge code. For example, in Figure 3, the orderable *Warfarin, 5 mg oral tablet *with an item number *17362344 *in the inpatient PM, had a charge code *7056345*, Multum drug code *d00022*, main Multum drug code (MMDC) *3616*, several NDCs *00056-0172-75 *(primary), *00832-1216-01*, etc. assigned to it. The charge code *7056345 *in the financial catalog was linked to item number *17362344 *in the inpatient PM, and using the MMDC *3616*, RxNorm code *855332 *could be assigned to it. Semantic attributes and links from RxNorm to other vocabularies could then be applied to the charge code *7056345*, thereby enriching the financial code. Any patient-level financial transaction referencing the charge code *7056345 *could be enriched using RxNorm, and cohorts of patients could be selected from financial data, using RxNorm.

### Integration of clinical data

**Figure 4:**
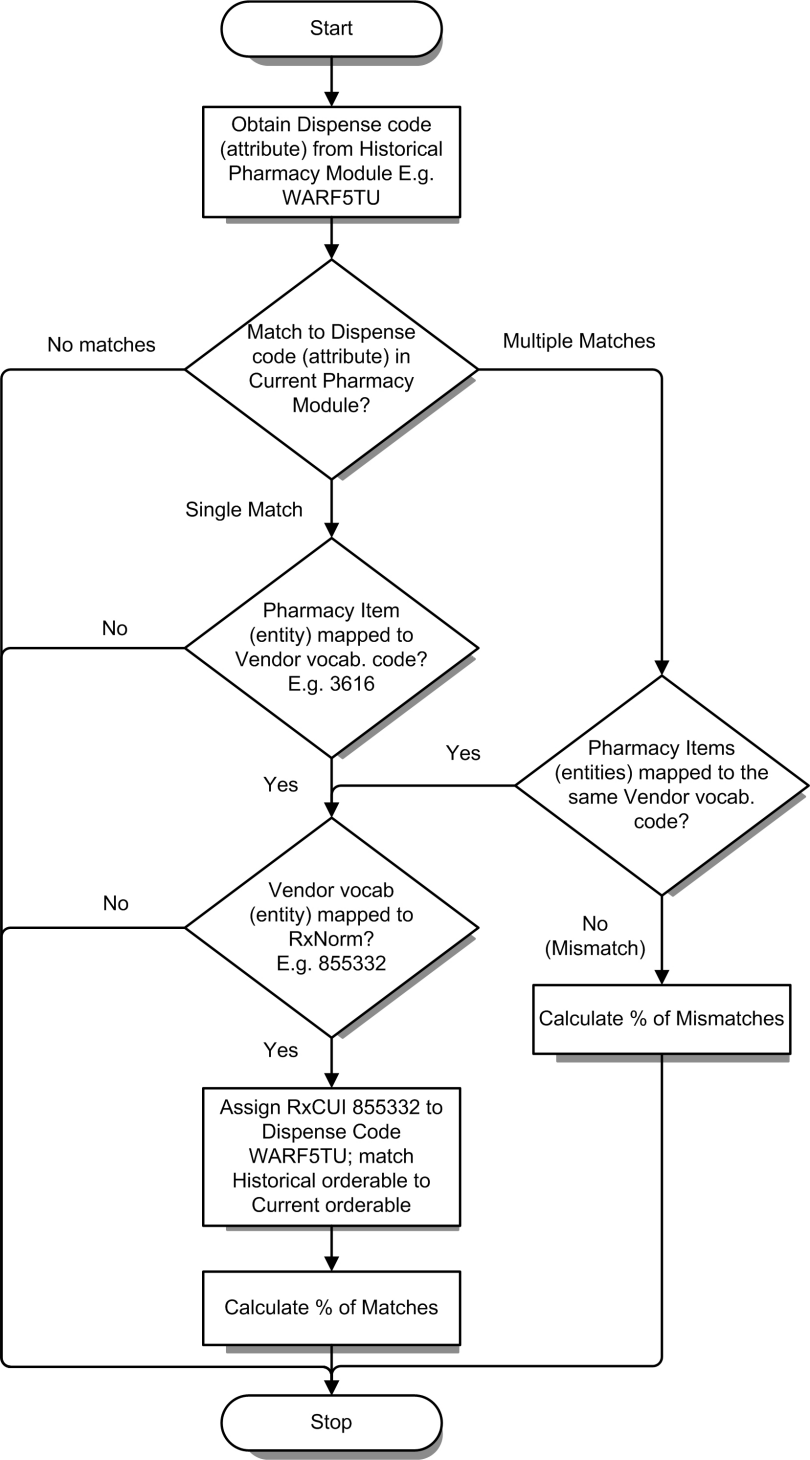
**Integration of historical clinical data**. An example of code-matching between the historical inpatient pharmacy module (PM) and the current inpatient PM, with links to RxNorm. *Warfarin, 5 mg oral tablet *in the historical inpatient PM had a dispense code of *WARF5TU*, which matched to a single item in the current inpatient PM. This item had the main Multum drug code (MMDC) *3616*, which mapped to RxNorm code *855332*, which could now be assigned to the historical code. Once the codes were matched across systems and mapped to RxNorm, a single query across historical and current data could be performed using RxNorm.

Integration of historical medication orderables with those in the current inpatient EHR was performed using either metadata attributes like dispense or charge code (Figure 2), or by using NDCs in combination with commercial vocabularies or RxNorm. RxNorm contained mappings to Multum based on generic names (GN), brand names (BN), semantic clinical drugs (SCD), and clinical drugs (CD), and to MediSpan based on CD. Integration of outpatient orderables was only possible using NDCs, since the outpatient system did not support some of the other metadata attributes which were available in the historical inpatient PM. Exact matches on the primary/representative NDC for each orderable were considered equivalent, and were mapped directly. In addition, for a given NDC in either of these catalogs, RxNorm, Multum and MediSpan were used to match by related NDCs using RxCUIs, MMDCs and GPIs respectively. In addition, Multum and MediSpan were also used as intermediaries when matching between orderables from various systems to RxNorm. For example, in Figure 4, the orderable *Warfarin, 5 mg oral tablet *in the historical inpatient PM had a dispense code of *WARF5TU*, which matched to a single item *17362344 *in the current inpatient PM. This item had the main Multum drug code (MMDC) *3616*, which mapped to RxNorm code *855332*, which could now be assigned to the historical code. Historical medication orders that used the dispense code *WARF5TU *could then be integrated with current inpatient orders, and cohorts containing both historical and current clinical data could be selected from both systems, using RxNorm.

### Evaluation using a research cohort

Inclusion criteria defined in an existing IRB approved study were used to evaluate the effect of the semantic enrichment process on cohort size for secondary use of EHR data based on the different methods for integration. The inclusion criteria consisted of patients who were prescribed warfarin, had a measurement of their International Normalized Ratio (INR) during the same visit as the medication order, and whose data were available in the EDW. The main outcome measure was cohort size obtained by using one or more integration and enrichment methods. For patients seen in an inpatient setting, warfarin is typically initiated while the patients are at the hospital, with subsequent follow-up being performed at a dedicated anticoagulation clinic in an outpatient setting, leading to their medication data being stored in two different EHR systems at our institution. The EDW contained records from different systems implemented and integrated during different time periods (Table 1), and the study was chosen among several others, since the medication part of the inclusion criteria spanned different systems, and because large sample sizes could be obtained due to warfarin being a highly prescribed drug.

## Results

### Characteristics of data sources

**Figure 5:**
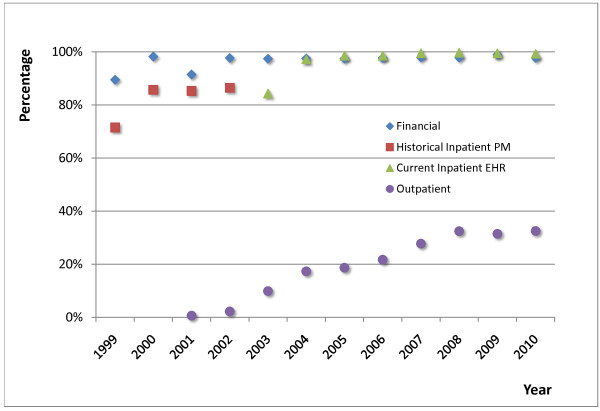
**Medication data by visits**. Percentage of encounters with medication data available in different systems. In the outpatient system, a single visit for a prescription refill or a lab test is considered a separate encounter, while the inpatient systems treat the entire inpatient stay as a single encounter; therefore, the percentage of encounters with medication data varies between the types of systems.

Until 2010, the current inpatient EHR contained medication records for over 175,000 inpatients, representing over 365,000 encounters, and over 6,200,000 medication orders. Upon including records from the historical inpatient system, the numbers exceeded 250,000 inpatients, with over 530,000 encounters, and over 7,200,000 medication orders. The outpatient EHR contained medication records for over 260,000 patients, representing over 2,150,000 encounters and over 4,000,000 medication prescriptions. Figure 5 shows the percentage of encounters with medication data available in the different source systems that were used in the study.

### Integration and enrichment of financial data

Table 2 shows a distribution of initial matches for the entire financial catalog, with snapshots from different years. The number of pharmacy items in the catalog grew consistently between the years, and a snapshot from 2009 included 5969 items, with 62.42% of the items matching charge codes in the current inpatient EHR. Mismatches, due to the assignment of the same financial charge code to two or more different drugs ranged from 0.69% to 0.73%. Among charge codes that had been in use, the most recent snapshot of the financial catalog contained 3226 codes and 94.33% of these items could be matched automatically with orderables in the current inpatient EHR, with a mismatch rate of 1.15%. Matches to the historical inpatient PM ranged from 40.74% - 48.9% for reference charge codes, and 61.28% - 75.82% for codes in use, using dispense codes for matching. Matches to the current outpatient EHR using primary/representative NDCs from the inpatient EHR ranged from 40.5% - 50.13% for reference charge codes, and 60.32% - 80.81% for codes in use. Matches to the outpatient EHR using Multum to find related NDCs were as high as 89.4% for charge codes used in 2009. Linking to vocabularies using NDCs resulted in up to 90.08% of charge codes that were used in 2009 being mapped to Multum, and up to 86.36% mapped to MediSpan using the primary NDCs from the inpatient EHR. Using related NDCs from Multum for the inpatient orderable resulted in a higher match of 90.02%. Charge codes enriched with RxNorm Clinical Drugs (CD) or Generic Names (GN) using Multum as an intermediate, ranged from 49.14% - 59.11% for the financial catalog to between 68.25% - 90.08% for codes that had been used in 2009.

**Table 2:** Integration of financial data

		Financial Catalog (complete reference)			Financial Catalog (codes used)		
		
Type	Match Description	1999 (N = 4532)	2004 (N = 5482)	2009 (N = 5969)	1999 (N = 2443)	2004 (N = 2638)	2009 (N = 3226)
Initial Match	Match to charge codes in current inpatient EHR	51.04%	55.76%	62.42%	73.41%	82.75%	94.33%
	
	(rate of mismatch)	(0.73%)	(0.73%)	(0.69%)	(1.11%)	(1.36%)	(1.15%)

Match to Clinical Systems	Clinical codes in current inpatient EHR by dispense codes	50.99%	55.67%	62.34%	73.33%	82.68%	94.27%
	
	Clinical codes in historical inpatient PM by dispense codes	47.26%	44.36%	40.74%	70.28%	75.82%	61.28%
	
	Clinical codes in outpatient EHR using primary NDC	40.29%	44.55%	50.13%	61.41%	70.43%	80.81%
	
	Clinical codes in outpatient EHR using related NDCs from Multum	46.89%	51.42%	57.01%	69.79%	79.19%	89.40%

Commercial Vocabularies	Multum drug codes	49.14%	53.65%	59.11%	71.52%	80.44%	90.08%
	
	Multum MMDCs	49.14%	53.65%	59.11%	71.52%	80.44%	90.08%
	
	MediSpan codes using primary NDCs from inpatient EHR	44.77%	49.53%	55.12%	66.91%	76.19%	86.36%
	
	MediSpan codes using related NDCs from Multum	48.37%	52.94%	58.44%	71.02%	80.10%	90.02%

RxNorm	RxNorm (CD) codes using primary NDCs from Inpatient EHR	46.34%	50.60%	55.59%	67.78%	76.23%	85.12%
	
	RxNorm (CD) codes using related NDCs from Multum	48.15%	52.57%	57.75%	70.20%	79.23%	88.38%
	
	RxNorm (CD) codes using Multum MMDCs	49.14%	53.65%	59.11%	71.52%	80.44%	90.08%
	
	RxNorm (GN) codes using Multum drug codes	49.14%	53.65%	59.11%	71.52%	80.44%	90.08%

Matching and semantic enrichment of financial catalogs from the different time periods yielded the numbers below.

### Integration of clinical data

Table 3 shows the percentage of items that matched between the historical inpatient system, the current outpatient system, and the current inpatient system by metadata and vocabulary based approaches. Matching by metadata-based approaches resulted in 84.44% match by charge codes for the reference catalog to 94.23% for the codes used in 2009. Matching by dispense codes resulted in fewer matches for the reference data (68.93%), although among codes that had been used, the matches ranged from 77.2% in 1999 to 94.68% in 2009. For vocabulary-based approaches, matching by primary NDCs ranged from 44.85% for reference data to 46.35% for codes that were still in use in 2009. Using Multum to find related NDCs, the rate of matching between the historical and current system was as high as 91.67%. Matching by primary NDCs to MediSpan and RxNorm was performed mainly to compare their baseline matches to those in Multum, and these were about 38.87% and 43.91% respectively. Using MediSpan and RxNorm to find related NDCs, the matches for codes that were still in use in 2009 were 62.89% and 70.37% respectively. Vocabulary based approaches for matching outpatient orderables using related NDCs from Multum, MediSpan and RxNorm resulted in a match of 17.8%, 20.63%, and 9.94%, respectively, for the reference, and 48.6%, 45.21% and 30.72%, respectively, for items that had been used in 2009.

**Table 3:** Integration of clinical data

				Medication Catalog (codes used)		
				
Source	Matching Approach	Matching Attribute	Medication Catalog (reference)	1999	2004	2009
Historical Inpatient System	Metadata	Charge Codes	2442/2892 (84.44%)	1858/2131 (87.19%)	1916/2155 (88.91%)	1618/1717 (94.23%)
		
		Dispense Codes	2622/3804 (68.93%)	1900/2461 (77.20%)	2037/2487 (81.91%)	1798/1899 (94.68%)
	
	Vocab.	Primary NDC (Multum)	1402/3126 (44.85%)	903/2151 (41.98%)	996/2260 (44.07%)	818/1765 (46.35%)
		
		Related NDCs (Multum)	2574 (82.34%)	1849 (85.96%)	1977 (87.48%)	1618 (91.67%)
		
		Primary NDC (MediSpan)	1129 (36.12%)	725 (33.71%)	844 (37.35%)	686 (38.87%)
		
		Related NDCs (MediSpan)	1835 (58.70%)	1234 (57.37%)	1360 (60.18%)	1110 (62.89%)
		
		Primary NDC (RxNorm)	1301 (41.62%)	850 (39.52%)	937 (41.46%)	775 (43.91%)
		
		Related NDCs (RxNorm)	2024 (64.75%)	1397 (64.95%)	1502 (66.46%)	1242 (70.37%)

Outpatient System	Vocab.	Primary NDCs	2786/39461 (7.06%)	432/1158 (37.31%)	1351/4285 (31.53%)	1734/6990 (24.81%)
		
		Related NDCs (Multum)	7023 (17.80%)	918 (79.27%)	2608 (60.86%)	3397 (48.60%)
		
		Related NDCs (MediSpan)	8140 (20.63%)	831 (71.76%)	2354 (54.94%)	3160 (45.21%)
		
		Primary NDC (RxNorm)	2611 (6.62%)	423 (36.53%)	1310 (30.57%)	1675 (23.96%)
		
		Related NDCs (RxNorm)	3921 (9.94%)	556 (48.01%)	1661 (38.76%)	2147 (30.72%)

Integration of orderables from historical inpatient and current outpatient to the current inpatient EHR system yielded the numbers below.

### Evaluation using a research cohort

**Figure 6:**
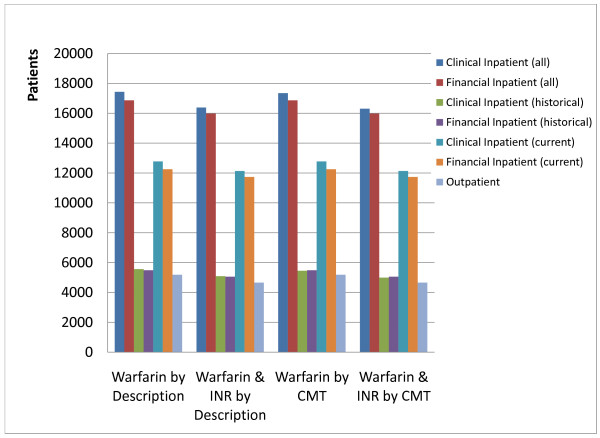
**Effect of data integration on cohort size**. Effect of matching and semantic enrichment techniques on cohort size of a retrospective study. INR: International normalized ratio; CMT: controlled medical terminology.

Figure 6 shows the effect of matching and semantic enrichment techniques on the size of the warfarin research cohort, while detailed results are provided in Additional File 1. In the absence of CMT, financial data could be queried using names/descriptions of medications; based on the inclusion criteria, cohorts of 15978 inpatients were obtained by queries based on both, CMT and descriptions. Searching by description in the clinical systems produced a cohort of 16386 inpatients, while searching by CMT produced a cohort of 16304 inpatients. Among inpatients, searching by description produced a cohort of 12128 in the current inpatient EHR, and 5087 in the historical PM, while searching by CMT produced cohorts of 12132 inpatients in the current inpatient EHR, and 4990 inpatients in the historical PM, among which, 4172 (25.58%) were found exclusively through the integration of historical clinical data. Searching in the outpatient system by description produced a cohort of 4653 patients, while searching by CMT produced a cohort of 4655 patients.

## Discussion

### Semantic enrichment of financial data

The semantic enrichment process for financial data leveraged common metadata attributes between the financial system and the current inpatient EHR, and used commercial vocabularies available in the EHR as links to RxNorm. The process of enrichment with CMT was limited by the availability of common metadata attributes within the current inpatient EHR, the consistent use of financial codes in the EHR, and also by links between the commercial vocabularies used in the EHR and CMT. Matches between the financial system and the EHR were significantly different for snapshots from the different years, with a 51.04% match in 1999, compared to a 62.42% match in 2009, even when the financial catalog itself was larger in 2009 than in 1999. Matching was also performed on a subset of financial codes that had actually been used for billing in the same years as the snapshots of the reference catalog, and these were substantially better, ranging from a 73.41% match in 1999 to 94.33% match in 2009. Although 94.33% of codes matched with items in the EHR in 2009, only 90.08% of the codes used in 2009 could be enhanced with CMT. In the inpatient pharmacy, certain items are mixed and dispensed or *compounded *in the hospital pharmacy, and such items may not always have single NDCs or MMDCs, since there could potentially be more than one drug involved. In such cases, reliable matches to CMT could not be obtained, and consequently, these were lower than the initial matches to the inpatient EHR.

The financial system is regarded as the source of truth for all financial data, and similarly, the clinical system is regarded as the source of truth for all clinical procedures, diagnoses, medication orders, etc. Automated charge capture processes (Figure 1) required the inclusion of financial charge codes in the current inpatient EHR as well as the historical PM, and this task was performed by expert pharmacists who maintained these systems over the years. The process of enrichment of financial data relied on the assumption that the copy of financial reference data that was maintained in the clinical system was a faithful representation of the original, i.e. the financial charge codes were appropriately assigned to the correct drug in the EHR. This assumption was tested during the matching process, so that a single charge code which matched with more than one MMDC (different drug, dose, route, form) was declared a mismatch. A mismatch rate of 0.69% (single charge code matched with multiple MMDCs) was found in the 2009 reference snapshot, while the rate was as high as 1.15% among financial codes that were used in 2009. While matching the entire financial catalog would have been ideal, the financial catalog itself was larger than the clinical catalog, since it contained multiple codes for the same drug, in order to facilitate processes like distribution of revenue among hospital service lines. In the absence of mappings to a CMT containing formal concept definitions, existing items in the financial catalog were likely duplicated when new ones were added, which contributed to the size, and introduced inconsistencies, which were identified in the form of mismatches. Ultimately, any strategy for enriching financial data would have to balance carefully the sensitivity afforded by the implicit level of trust between copies of reference metadata in different systems, and the specificity afforded by defining rules for estimating the quality of the reference information.

### Integration of clinical data

Each of the two other EHR systems considered in the investigation had different metadata and vocabulary elements in common with the current inpatient EHR (Table 1 and Figure 2). The historical PM had supported Multum, and the best way to integrate it with data from the current EHR would have been using Multum itself; however, Multum codes were not included in the final reference snapshot that was taken before the historical system was decommissioned, and these codes were not present in the archived HL7 messages sent to the dispensing system, which contained only the dispense codes. In addition, the purpose of this investigation was to develop more generic methods for integration of historical data, so other metadata and vocabulary elements (e.g. charge code, dispense code, NDCs) were preferred. Although NDCs existed in the historical PM, many of them did not have direct matches with NDCs in the current inpatient system, possibly due to certain codes becoming obsolete, and direct matches were limited to 44.85% in the reference catalog to 46.35% in the 2009 snapshot of codes in use even after normalizing the historical NDC format to 11-digits. Multum, MediSpan and RxNorm were also used to find related NDCs for the primary NDCs from the historical system in order to improve matching. Not surprisingly, matching on related NDCs using Multum produced the best results--with 82.34% match on reference catalog and 91.67% match in the 2009 snapshot--since the historical system had used Multum at one point, and many of these NDCs would have existed in older versions of Multum. Relying on metadata attributes like charge and dispense codes from the historical system produced matches as high as 84.44% and 68.93% respectively for the reference snapshot, and 94.23% and 94.68% respectively for codes used in 2009.

The link by dispense codes was noteworthy because these codes uniquely identified the items between the historical system and the dispensing cabinets, and even at 68.93%, they represented 2622 matching items, which was higher than the number obtained by any other method, including matches by charge code (2442), related NDCs using Multum (2574), RxNorm (2024) or MediSpan (1835) as a reference. Since the current EHR as well as historical PM communicated with the dispensing system using HL7 messages that contained dispense codes, the dispensing systems served as the source of truth for dispense codes, whereas both the current and historical inpatient systems contained copies of these codes to facilitate HL7 messaging between systems, in a manner similar to having copies of charge codes from the financial system. Charge codes were also used similarly to create matches between the different systems, and produced matches as high as 94% between the two different inpatient systems. Ideally, matches between different systems should be performed using concepts from a CMT or pharmacy vocabularies. The high percentages of matches obtained using metadata-based methods suggest that if pharmacy vocabularies or CMT were not available in the different systems, then metadata references to a common, external system could be used as a substitute to perform initial matches between different systems, which can then be reviewed by experts.

### Centralized Vs. decentralized metadata

In both instances of integration described above, it was observed that integration using administrative metadata such as charge and financial codes was either better than or as good as integration using CMT. These findings can be explained by differences in how the different systems handle each of these data elements, and vocabularies. Centralized metadata and vocabulary management solutions have been proposed as solutions for enterprise-wide knowledge management, and several successful implementations exist across the nation. Unlike a centralized metadata management solution which can serve as the single source of truth for all metadata attributes referenced by other systems, metadata elements in the present investigation were obtained from different systems. Under this decentralized model, the financial system served as the source of truth for charge codes, the automated dispensing system served as the source of truth for dispense codes, the current inpatient EHR system served as the source of truth for current inpatient formulary items, and so on. No single system served as a centralized metadata store, although the current inpatient system contained the highest number of metadata and vocabulary elements among the systems considered in this investigation.

Exchange of data between these systems (Figure 1) using messaging standards like HL7 relies on coded data elements such as charge codes, dispense codes and catalog codes used to identify different medication items. Within each system which serves as the source of truth for that particular data element (Figure 2), the discrete unit is an *entity*, and each entity can have *attributes *in the form of reference to entities in *other *systems. For example, a charge code is an entity within the financial system, but an *attribute *of an entity *item *within the current inpatient EHR and historical inpatient PM, so that those systems can post transactions in the financial system by using the charge code as a token. Similarly, a dispense code is an *entity *within the dispensing system, but is an *attribute *of an entity *item *in the current inpatient EHR and historical inpatient PM. Even with changes in the EHRs, attributes with references to foreign systems changed, but the entities themselves remained intact in other systems. Consequently, data from EHRs, which referred to common *entities *in other systems, could be easily integrated using such metadata. Although various components of the financial and clinical infrastructure were replaced over a period of time (Table 1), at least one or more of the systems which served as sources of truth had continued to be in use, so that the changes themselves were staggered across a period of time. In addition, both data and metadata from each of these systems were available in the EDW, which served as a historical, longitudinal reference, as well as the venue for data integration. In order to successfully use metadata from different systems in a heterogeneous environment for data integration, systems that serve as sources of truth would have to meet the above criteria for persistence, and historical data and metadata, such as those typically stored in an EDW would have to be readily available.

### Evaluation of the enrichment process

Retrospective research using archived data requires quality data, as well as a sound understanding of how those data were captured in the EHR. The selection of patient cohorts for retrospective research, or even the enrollment of new patients in prospective studies would require reliable identification based on the inclusion or recruitment criteria. While it may be possible to search for cohorts from financial data based on descriptions of various *line items *in the patient's bills, financial descriptions are often based on institutional needs, and in order to enable sharing and consistent reporting of these data, it would become necessary to adopt common vocabularies. Enriching financial data with CMTs like RxNorm can allow for consistent, normalized and semantically accurate description of financial data, which can also be shared outside a given institution. Inclusion criteria from an IRB approved study, consisting of patients who were prescribed warfarin and had INR measurements performed during the same visit were used to obtain estimates of cohort sizes (Figure 6 and Additional File 1). Cohorts obtained from the financial system were slightly smaller than those obtained through clinical systems, due to the matching methods used, although it was noteworthy that 98% of the patients in the cohort identified from the clinical systems could also be identified using financial data. Since the financial system supported automated charge capture as well as manual charge entry processes from several source clinical systems (Figure 1), it would contain charges for items from multiple systems, which may or may not have corresponding matches to specific items in the inpatient EHR systems. Consequently, one might expect to find more patients in the financial system, than in the clinical system, although this was not observed in our investigation. In the absence of consistently used charge code, with meaningful descriptors in the financial system, manually billed items could have been assigned different codes than would otherwise be assigned in an automated charge capture process from the inpatient EHR. The FS also contained charge codes for *miscellaneous pharmacy items*, which can potentially create the same types of problems as not elsewhere classified (NEC), or not otherwise specified (NOS) [4], in vocabularies which are not true CMTs. Regardless of the source for obtaining the research cohort using automated methods, a more in-depth manual chart review would need to be performed on cohorts obtained using automated methods.

### Implications of the findings

The transition to and implementation of EHR systems can often be a phased, multistep process, which can span several years. During the transition to EHRs, replacement of existing EHRs with newer systems, or with the gradual addition of functionality to an existing EHR, through functional integration with other systems, the type and richness of captured data can change or evolve. For institutions that have transitioned from older systems to modern EHRs, a large amount of data may still exist in older, semantically poorer formats, or such data may only be available from financial and billing systems, rather than clinical systems. Commercial EHRs are more pervasive than those developed *in-house*, and among commercial EHRs, it is rare to find support for third-party centralized metadata repositories or vocabulary services that can serve as sources of CMTs. Many commercial EHRs, instead, rely on their own solutions for managing metadata and vocabularies, which may be internally consistent within the systems, but difficult to integrate with analogous components of other, external systems. Given EHR implementations that employ a *best of breed *approach for different components such as pharmacy, labs, radiology, etc., such components may be derived from different commercial applications, with different approaches to the management of metadata and vocabularies, and centralized metadata and vocabulary services may not be feasible, thus creating data integration challenges that could potentially undermine some of the benefits of having EHRs.

Metadata-based methods developed in this investigation relied on the assumptions that certain systems were reliable, persistent sources of truth for specific metadata elements, that the copies of metadata elements in external systems were faithful representations of the original, and that these copies had been properly assigned as attributes of entities in those systems (Figure 2). Vocabulary-based methods developed in this investigation worked on similar assumptions that external vocabularies had been implemented and used properly in the different systems.

**Figure 7:**
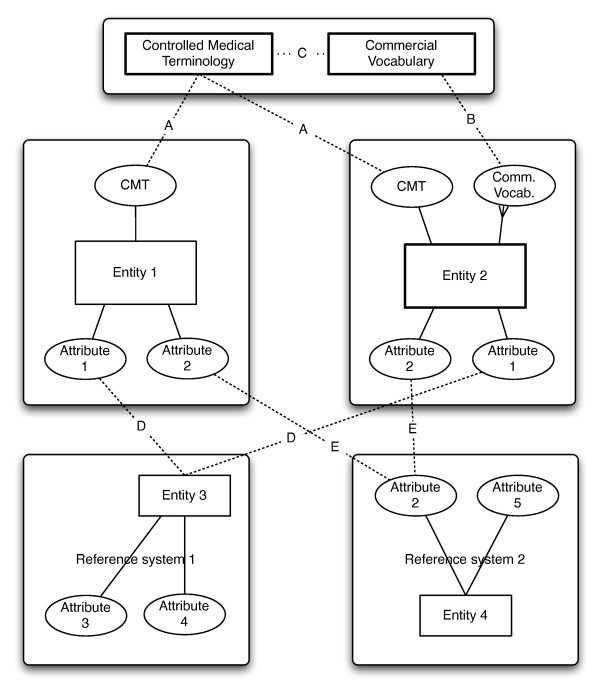
**Generalize the integration approaches**. Consider two generic electronic health record (EHR) systems with medication orderables: Entity 1 and Entity 2. If both entities were mapped to controlled medical terminologies (CMT), ontological matching could be achieved through CMT (path A). If one of the two systems were to only contain mappings to a commercial vocabulary, then matching could still be accomplished through path B, assuming that the commercial vocabulary had been mapped to a CMT (path C), which would then allow vocabulary-based matching between Entity 1 and Entity 2. If CMTs were not available, but the systems had common metadata attributes (e.g. Attribute 1), which could be validated against an entity (Entity 3) in an external reference system (Reference System 1), then metadata-based matching is possible between Entity 1 and Entity 2 through path D. Matching could also be performed if the systems had other metadata attributes in common (e.g. Attribute 2), which matched with an attribute in another reference system (path E), as could direct matching between the two, although the latter approach would be less than ideal.

Both of these approaches could be generalized and applied to other commercial systems, using different vocabularies and metadata attributes, as illustrated in Figure 7. Consider two generic electronic health record (EHR) systems with medication orderables, 'Entity 1' and 'Entity 2.' If both entities contained mappings to CMT, exact ontological matching could be achieved (path A). If one of the two systems were to only contain references to a commercial vocabulary, then matching could still be accomplished through path B, assuming that the commercial vocabulary had been mapped to the CMT (path C). On the other hand, if CMTs were not available, but the systems had metadata attributes in common (Attribute 1), which could be validated against an entity (Entity 3) in an external reference system (Reference System 1), then metadata-based matching could be performed through path D. Matching could also be performed if the systems had other metadata attributes in common (e.g. Attribute 2), which had a match to an attribute in another reference system (path E), as could direct matching between the two, although such an approach would be less than ideal.

None of the methods under either approach assumed centralized metadata and vocabulary models, but rather relied on identifying and designating external, persistent stores of metadata and vocabularies as sources of truth. Inasmuch as EHR infrastructures in different settings are able to satisfy the above conditions, the above methods can be generalized to those settings. The ultimate implications of these findings in the context of the state of EHR implementations are that financial medication data can be repurposed for research through semantic enrichment techniques. In the absence of consistently used pharmacy vocabularies in historical/legacy data, automated metadata-based methods can be used for data integration, and a combination of both techniques would allow the creation of large, longitudinal datasets which can be used in research.

### Limitations

Due to differences in billing processes between the inpatient and outpatient EHRs, the enrichment of financial data, and it's comparison to clinical data was limited to data collected through the inpatient system. In addition, although the outpatient system supports CPOE, a dedicated outpatient pharmacy module has not been implemented at our institution; therefore, direct formulary to formulary comparisons between the inpatient and outpatient systems were not possible at the time of this investigation. Finally, our choice of using cohort selection in a retrospective study was motivated by the need to investigate differences in the approaches and to examine if enriched financial data may be suitable for this purpose, and before broadly applying these findings, an investigator may need to assess these processes using examples that resemble their potential use cases.

## Conclusions

EHRs evolved gradually from more pervasive specialized hospital information systems over a period of time. With HITECH meaningful use requirements, as more hospitals adopt modern EHRs, financial and historical clinical data will remain abundant, and the consolidation, enrichment and integration of such data have the potential for creating large sets of data that can be used for selecting cohorts in retrospective studies, or potentially recruit patients for prospective studies. Commercial pharmacy vocabularies used in EHRs, which map to CMTs like RxNorm, compared favorably with RxNorm for integration of data between different clinical systems. Metadata-based methods for data integration performed as well as or at times better than vocabulary-based methods. Using metadata from different systems which served as sources of truth for those metadata elements in a decentralized manner was successful due to the staggered replacements of components in the EHR infrastructure, which allowed for persistence of metadata in different systems. In the absence of common vocabularies in different systems, metadata-based approaches could potentially be used for matching and data integration. For institutions that are transitioning to modern EHRs, the development of strategies for integration and enrichment--such as the ones described in this study--could allow repurposing of historical data for research.

## Abbreviations

A complete list of abbreviations used in this manuscript has been provided in Additional File 2.

## Competing interests

The authors have no competing interests to declare.

## Authors' contributions

VGD conceived and planned this study as part of a larger body of work for his dissertation, along with his advisor JAM. NBS and CYH provided expertise in pharmacy automation systems and data warehousing respectively. All authors read and approved the final manuscript.

## Pre-publication history

The pre-publication history for this paper can be accessed here:

http://www.biomedcentral.com/1471-2288/11/151/prepub

## Supplementary Material

Additional file 1 **Effect on sample size of a research cohort**. Unlike inpatient medication *orders*, outpatient medication data for Warfarin mainly consists of *prescriptions *which do not post charges in the financial system automatically (refer to Figure 1). A single recurring medication order can post multiple transactions in the financial system for each subsequent instance of the order; consequently, there were more transactions in the financial system than there were orders in the clinical system. Click here for file

Additional file 2 **List of abbreviations**. List of different standard and local abbreviations, used in the manuscript. Click here for file
