# Supplementary material for: Integrating historical clinical and financial data for pharmacological research
Source: BMC Med Res Methodol. 2011 Nov 18;11:151. doi: 10.1186/1471-2288-11-151 (PMC3252280; doi:10.1186/1471-2288-11-151)
Supplement: Additional file 1 — Effect on sample size of a research cohort. Unlike inpatient medication orders, outpatient medication data for Warfarin mainly consists of prescriptions which do not post charges in the financial system automatically (refer to Figure 1). A single recurring medication order can post multiple transactions in the financial system for each subsequent instance of the order; consequently, there were more transactions in the financial system than there were orders in the clinical system. [file 1471-2288-11-151-S1.DOC]

**Additional File I**: Effect of vocabulary-matching and semantic enrichment on cohort sizes. Unlike inpatient medication *orders*, outpatient medication data for Warfarin mainly consists of *prescriptions* which do not post charges in the financial system automatically (refer to Figure 1). A single recurring medication order can post multiple transactions in the financial system for each subsequent instance of the order; consequently, there were more transactions in the financial system than there were orders in the clinical system.

| Cohort Selection Method | System | Clinical Data | | | Financial Data | | |
| --- | --- | --- | --- | --- | --- | --- | --- |
|  |  | Patients | Encounters | Orders /  Prescrip. | Patients | Encounters | Transactions |
| All Transact. | Inpatient | 258873 | 536648 | 7231621 | 292413 | 641913 | 35835838 |
|  | Histor. (I) | 104793 | 171953 | 999647 | 108109 | 178891 | 8676556 |
|  | Current (I) | 175707 | 365395 | 6242669 | 208511 | 463961 | 27185600 |
|  | Outpatient | 261750 | 2172237 | 4014826 | - | - | - |
| Patients with INR & Medications | Inpatient | 55468 | 90443 | 3325635 | 57516 | 95184 | 15722554 |
|  | Histor. (I) | 13215 | 19331 | 701723 | 13339 | 19409 | 2698533 |
|  | Current (I) | 44748 | 71226 | 2626265 | 46739 | 75896 | 13024021 |
|  | Outpatient | 21339 | 515444 | 1024334 | - | - | - |
| Warfarin by Description | Inpatient | 17438 | 29126 | 78509 | 16867 | 27833 | 169542 |
|  | Histor. (I) | 5558 | 8615 | 24611 | 5485 | 8461 | 49704 |
|  | Current (I) | 12776 | 20538 | 53919 | 12247 | 19401 | 119871 |
|  | Outpatient | 5184 | 22275 | 23927 | - | - | - |
| Warfarin & INR by Description | Inpatient | 16386 | 26583 | 75308 | 15978 | 25598 | 160802 |
|  | Histor. (I) | 5087 | 7562 | 23226 | 5053 | 7471 | 45525 |
|  | Current (I) | 12128 | 19037 | 52092 | 11731 | 18143 | 115277 |
|  | Outpatient | 4653 | 21591 | 23186 | - | - | - |
| Warfarin by CMT | Inpatient | 17345 | 28962 | 75113 | 16867 | 27832 | 169535 |
|  | Histor. (I) | 5452 | 8457 | 22931 | 5485 | 8460 | 49697 |
|  | Current (I) | 12770 | 20521 | 52182 | 12247 | 19401 | 119871 |
|  | Outpatient | 5187 | 22280 | 23932 | - | - | - |
| Warfarin & INR by CMT | Inpatient | 16304 | 26441 | 71948 | 15978 | 25598 | 160802 |
|  | Histor. (I) | 4990 | 7419 | 21561 | 5053 | 7471 | 45525 |
|  | Current (I) | 12132 | 19036 | 50387 | 11731 | 18143 | 115277 |
|  | Outpatient | 4655 | 21595 | 23190 | - | - | - |
