# Supplementary material for: Integrating historical clinical and financial data for pharmacological research
Source: BMC Med Res Methodol. 2011 Nov 18;11:151. doi: 10.1186/1471-2288-11-151 (PMC3252280; doi:10.1186/1471-2288-11-151)
Supplement: Additional file 2 — List of abbreviations. List of different standard and local abbreviations, used in the manuscript. [file 1471-2288-11-151-S2.DOC]

**Additional File II**: List of Abbreviations.

BN Branded Name (RxNorm)

CC Charge Code

CD Clinical Drug (RxNorm)

CMT Controlled Medical Terminology

CPOE Computerized Provider Order Entry

CPT® Current Procedural Terminology

DC Dispense Code

DRG Diagnosis Related Group

DS Dispensing System

EDW Enterprise Data Warehouse

EHR Electronic Health Record

eMAR Electronic Medication Administration Record

FS Financial System

GN Generic Name (RxNorm)

GPI MediSpan® Generic Product Identifier

HCPCS Healthcare Common Procedure Coding System

HITECH USA Health Information Technology for Economic and Clinical Health Act 2009

HL7 Health Level Seven

ICD9-CM International Classification of Diseases Ninth revision, Clinical Modification

INR International Normalized Ratio

IRB Institutional Review Board

MMDC Main Multum® Drug Code

NDC National Drug Code

NEC Not Elsewhere Classified (ICD9-CM)

NOS Not Otherwise Specified (ICD9-CM)

PM Pharmacy Module

RxCUI RxNorm Concept Unique Identifier

SCD Semantic Clinical Drug (RxNorm)

SNOMEDCT Systematized Nomenclature of Medicine Clinical Terms
